# Primiparous and Multiparous Women’s Mode of Birth and Negative Emotions

**DOI:** 10.3390/ijerph19095189

**Published:** 2022-04-25

**Authors:** Gizell Green, Riki Tesler, Adilson Marques

**Affiliations:** 1 Nursing Department, Ariel University, Ariel 40700, Israel; 2 Department of Health Systems Management, School of Health Sciences, Ariel University, Ariel 40700, Israel; riki.tesler@gmail.com; 3 Centro Interdisciplinar do Estudo da Performance Humana, Faculdade de Motricidade Humana, Universidade de Lisboa, Estrada da Costa, 1499-002 Cruz Quebrada, Portugal; adncmpt@gmail.com; 4 Instituto de Saúde Ambiental, Faculdade de Medicina, Universidade de Lisboa, 1499-002 Lisbon, Portugal

**Keywords:** childbirth, negative emotions, primiparous, multiparous, mode of childbirth

## Abstract

Negative childbirth experiences may result in negative emotions that may lead to negative outcomes, such as post-traumatic stress disorder. We aimed to examine the differences in emotions between primiparous and multiparous women and mode of birth. We used a retrospective cross-sectional study design with three hundred and fifty women. Primiparous women reported higher levels of fear, lack of control, and dissociation emotions compared to multiparous women. The EmCs (emergency cesarean section) group experienced the most fear, lack of control, anger, and conflict emotions. It is important to conduct follow up work with women who underwent unplanned birth procedures since negative childbirth experiences may lead to further negative effects on women’s psychosocial health and well-being.

## 1. Introduction

Giving birth to a child is a process that is accompanied by both happiness and pain [1]. Labor and childbirth are significant experiences for most women and may trigger a wide range of emotions during and after childbirth. Although childbirth is a natural biological process, it is characterized by a far-reaching and predictive physiological series of events. A woman’s subjective experience and the specific mode of childbirth are often interrelated and deeply personal. On the one hand, a positive childbirth experience can have a long-lasting effect on women’s self-efficacy and self-esteem [2]. On the other hand, a negative childbirth experience may lead to negative outcomes, such as fear of childbirth, maternal distress, depression, and post-traumatic stress disorder (PTSD) [2,3,4]. 

Previous research has shown that a high percentage of women (between 20% to 48%) who perceived their childbirth to be traumatic later reported symptoms of PTSD [3,5]. In order to reduce the fear of childbirth in women, it is necessary to identify the factors contributing to it [1]. Negative emotions during childbirth, such as fear, lack of control, anger, conflict, dissociation, fault, and failure, were found to be the prominent influencing factors that may have caused postpartum PTSD [6,7,8]. Fear; lack of control; and dissociation, which refers to interrupting or detaching from a psychological function such as identity, memory, or perception [9], were the most prominent factors for PTSD [8]. Women’s childbirth characteristics, such as obstetric background (primiparous or multiparous) and childbirth mode (planned or unplanned procedures), are known catalysts for negative emotions during childbirth. 

Primiparity refers to giving birth for the first time, which often translates into feelings of inexperience and an inability to assess the childbirth experience. Thus, primiparous women are more likely to feel uncertainty and fear related to childbirth than multiparous women (i.e., women who have given birth in the past) [10]. Among multiparous women, previous birth experiences may influence their emotions during the next childbirth. A study showed that the mean fear of childbirth was higher in primiparous women compared to multiparous women. Moreover, the mean childbirth self-efficacy in primiparous women was significantly lower than that of multiparous women [1]. In addition, other previous studies found that fear of childbirth was higher in primiparous women than in multiparous women [11,12]. However, there are researchers with different results. It has been found that a higher rate of multiparous women, as compared to primiparous women, reported acute fears of childbirth [13,14]. The fears of multiparous women in comparison with primiparous women might be the result of a previous traumatic childbirth and this suggests that they suffered from post-traumatic stress disorder (PTSD) postnatal care [15]. Due to the inconsistency of findings, it is important to further investigate aspects that relate to emotions during childbirth in primiparous women compared to multiparous women. This is especially true in cases where childbirth progression is unknown or unexpected [16]. One previous study showed that women who experienced negative vaginal births feared future vaginal birth, potentially generating negative emotions and fear during labor [17]. Fertility levels in Israel are higher in relation to other developed countries [18]. Therefore, it is important to investigate previous childbirth experiences and emotions towards childbirth in all countries, especially those with high levels of fertility. In addition, the mode of birth (e.g., vaginal birth [VB], vacuum extraction, and planned or emergency cesarean) can influence women’s emotions.

This study refers to VB as the natural and most common mode of birth. Other modes of birth may involve intensive obstetric intervention, including vacuum extraction (VE) and elective (ElCs) or emergency cesarean section (EmCs). Studies have shown that the lower the number of obstetric interventions, the greater the odds for women’s positive birth experiences and vice versa [19,20]. Obstetric intensive interventions can contribute to a traumatic experience [21], which can cause negative emotions. Additional studies confirmed that women expressed contentment during birth when they exhibited more involvement in decision making regarding the mode of birth and vice versa [22]. Accordingly, it is important to delineate which specific negative emotion corresponds to which mode of birth. A crucial factor that can affect a woman’s emotions during birth is whether the birth is conducted through planned or unplanned procedures. 

Women who underwent planned birth procedures (VB or ElCs) reported significantly more positive emotions during childbirth than those who underwent unplanned birth procedures (VE or EmCs) [2]. Another study found that women who had spontaneous vaginal births reported the most positive emotions regarding their birth, while the women who had had unplanned cesarean births were at the least positive [23]. Another study found that women who had had a spontaneous vaginal birth reported the most positive feelings about their birth, while the women who had had an unplanned cesarean birth were the least positive [24]. The last group may feel disappointed, upset, sad, angry, and like a failure. Thus, the more a woman was acquainted with the birth process, the more she felt in control and satisfied during the process. However, if the birth took an unplanned turn and obstetric intervention was required, their emotions were more likely to be negative. 

**Figure 1:**
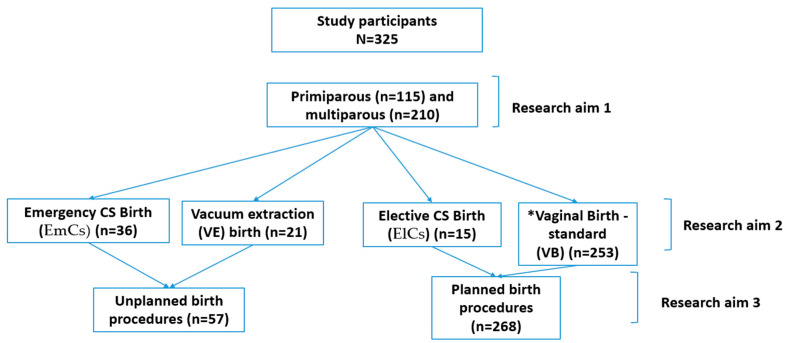
Participants and research aim flow diagram. Abbreviation: * the study used terminology of vaginal birth instead of standard vaginal of delivery.

Accordingly, it is important to examine factors such as primiparous and multiparous women, mode of birth, and planned’ birth versus ‘unplanned’ birth procedures and their potential to be associates with negative emotions in birth. Since negative emotions during birth may cause negative consequences, such as PTSD and depression, in the short and long term [2,3]. It may allow us to make intervention programs according to this factor. Hence, the study aims are (See Figure 1):

1. To compare the negative emotions of primiparous and multiparous women during their last birth;
2. To compare the differences between the negative emotions during birth based on the mode of birth: VB, VE, ElCs, and EmCs;
3. To compare the negative emotions between women that experienced ‘planned’ birth procedures compared to ‘unplanned’ birth procedures.

## 2. Materials and Methods

### 2.1. Study Design and Procedure 

We applied a retrospective, cross-sectional comparison study. The study was conducted in the State of Israel. We contacted online social networks with potential volunteers through a massage requesting to complete the questionnaire through the internet. Those who wanted to volunteer for the research sent their email address to receive a link to the questionnaire. The questionnaire was then delivered and took an estimated time of approximately 10 min to fill out. The participants did not receive any compensation for participation in the research. The e-mail was sent to approximately 1493 potential applicants, and 325 expressed interest and participated in the study. The overall response rate for research participation was 22%, which is a good response rate.

### 2.2. Participants

Three hundred and twenty-five women participated in the research. The inclusion criterion included having given birth in the two previous years, being free from pregnancy and obstetric complications (such as chronic health disorders, third- or fourth-degree perineal tear, and neonatal abnormalities), and multiple pregnancy. The exclusion criterion was women who never gave birth. 

### 2.3. Ethical Considerations

The University Institutional Review Board approved this research (approval number AU-HEA-GG-20190814-1). The participants enrolled voluntarily, were informed of the research aims, and signed electronic informed consent before filling out the questionnaire. The participants were also informed about the option to withdraw from the study at any point, that their questionnaire responses would remain confidential, and that the data would be analyzed discretely. 

### 2.4. Instruments

To assess women’s emotions and their relation to birth details and mode of birth, the questionnaire was comprised of three sections. The first section referred to participants’ background characteristics (e.g., age, religion, and parity). The second section included obstetric background (i.e., number of previous births and mode of last birth (VB, VE, EMCS). The third part of the questionnaire included categories describing personal cognitions and emotions of intrapartum hotspots, referring to moments of extreme distress during traumatizing events. The categories contained several emotions: fear and lack of control (example of item: during the last birth you felt out of control of what was going on), which included six items (the internal reliability in this study was α 0.91); anger and conflict (example of item: during the last birth you felt angry), which included five items (the internal reliability in this study was α 0.80); intrapartum dissociation, (example of item: during the last birth you felt detached as if in a dream), which included five items (the internal reliability in this study was α 0.84); and failure and fault (example of item: during the last birth you felt guilty), which included three items (the internal reliability in this study was α 0.84). The total responses ranged from 0 = “not at all” to 3 = “extremely” [7]. The survey was translated from English to Hebrew and vice versa and validated. 

### 2.5. Data Analysis

A statistical analysis was performed using the Statistical Package for the Social Sciences (SPSS TM), 25.0 version, Chicago, IL, USA. A parameters analysis test was performed since the groups were not equal in size. In addition, we conducted the Cronbach’s alpha, descriptive statistics, Mann-Whitney non-parametric analysis as an independent sample test, Spearman correlation, and Kruskal-Wallis one-way analysis of variance.

## 3. Results

The participant characteristics are presented in Table 1. The mean age of the participants (*n* = 325) was 28.79 ± 5.39 years. Most of the women were religious (151; 46%), married (309; 95%), had a higher education (284; 87%), and had one child (210; 65%). 

For examining research aim 1, we conducted a Mann-Whitney non-parametric analysis as an independent sample test and found that primiparous women reported higher levels of fear, lack of control, and intrapartum dissociation emotions than multiparous women during their previous birth. Regarding anger and conflict and failure and fault, no differences were found between the groups (Table 2). 

First, correlations were found between all variables related to negative emotions (fear and lack of control, anger and conflict, intrapartum dissociation, and failure and fault). In addition, one meaningful negative correlation was found between the number of children birthed and the emotion of intrapartum dissociation (r = −0.32, *p* < 0.00). Hence, the higher the number of children, the less the woman felt intrapartum dissociation. The other two relationships of number of children birthed with fear and lack of control (r = −0.16, *p* < 0.00) and failure and fault (r = −0.11, *p* < 0.00) were very weak and, thus, insignificant (Table 3).

For examining research aim 2, we conducted a non-parametric Kruskal-Wallis one-way analysis of variance and found significant negative emotion differences, including fear and lack of control, anger and conflict, intrapartum dissociation, and failure and fault during birth based on the mode of birth (VB, VE, ELSC, or EmCs; χ^2^ (3) = 33.27, *p* < 0.00; χ^2^ (3) = 12.16, *p* < 0.00; χ^2^ (3) = 11.22, *p* < 0.01; χ^2^ (3) = 56.56, *p* < 0.00, respectively). The group that felt the most fear and lack of control and anger and conflict emotions was the EmCs group, as compared to the VB group. The group that felt the most intrapartum dissociation emotions was the VE group; the VB group experienced these emotions the least. The group that felt the most failure and fault emotions was the ElCs group; the VB group experienced these emotions the least (Table 4).

For examining research aim 3, we conducted a Mann-Whitney non-parametric analysis as an independent sample test and found that there were significant differences between the two groups regarding planned vs. unplanned birth procedures. Women who experienced unplanned births procedures reported higher levels of fear and lack of control (z = 4.89, *p* < 0.00), anger and conflict (z = 2.13, *p* < 0.03), and fault and failure (5.41, *p* < 00) than women who underwent planned procedures (Table 5). 

## 4. Discussion

The present study strived to compare the emotions of primiparous and multiparous women during their previous birth to compare negative emotion differences during birth based on the mode of birth (VB, VE, ElCs, and EmCs), and to compare negative emotions between women that experienced ‘planned’ birth versus ‘unplanned’ birth procedures. According to the results of the present study, primiparous women reported higher levels of fear, lack of control, and intrapartum dissociation emotions than multiparous women during their previous birth. Additionally, the group that felt the most fear, lack of control and anger, and conflict emotions was the EmCs group, as compared to the VB group. The group that felt the most intrapartum dissociation emotions was the VE group; the VB group experienced these emotions the least. The group that felt the most failure and fault emotions was the ElCs group; the VB group experienced these emotions the least. Furthermore, significant differences between the two groups regarding planned vs. unplanned birth procedures were found. Women who experienced unplanned birth procedures reported higher levels of fear and lack of control, anger and conflict, and fault and failure than women who underwent planned birth procedures.

Like other studies, we found that primiparous women feared more for their health and life than multiparous women. One study demonstrated that primiparous women, as compared to multiparous women, felt more fear during birth. Additionally, primiparous women had significantly lower self-efficacy compared to multiparous women during childbirth [1]. Moreover, verbal violence from the caregiver’s crew can cause fear during the birth and is also associated with the appearance of PTSD [25]. Hence, appropriate verbal treatment and the provision of concrete and understandable information as well as ensuring informed consent can serve as elements that could help reduce fear during childbirth [26], especially regarding primiparous women. On one hand, our study results correspond with other prior studies that found that fear of birth was higher in primiparous women than in multiparous women [11,12]. However, on the other hand, our findings are dissimilar to other studies that found a higher mean of multiparous women, as compared with primiparous women, that reported high levels of fear of giving birth [13,14]. The last two studies assumed that the fears of multiparous women compared with primiparous women may be the result of a previous traumatic birth and suggested that they suffered from post-traumatic stress disorder (PTSD) postpartum care [15]. Therefore, it is important to further examine issues related to feelings during birth in primiparous women compared to multiparous women since a meta-analysis revealed that the risk factors most strongly associated with postpartum PTSD included negative subjective birth experiences and dissociation [27]. Another study of RCT conducted among primiparous women given that the fear of childbirth can affect the choice of birth method, measuring the level of fear and anxiety and determining the level of self-confidence and self- efficacy of women in pregnancy can help members of the healthcare team to identify those women who request cesarean section out of fear or anxiety [24]. To decrease these emotions, it is important to attend primiparous women, discuss their expectations, and design more informative interventions for them [16].

The group that felt the most fear, lack of control, anger, and conflicting emotions was the EmCs group. These findings are in line with another study that found that EmCs was a strong predictor of negative birth experiences [2]. An additional study found that more than half of women who experienced an EmCs reported emotions such as an intense fear of death and injury to themselves or their baby during the birth procedure [28]. Feelings such as lack of control can be linked to future complications, including PTSD [27]. Moreover, the group that felt the most dissociation was the VE group. It is important to emphasize this issue and take care of women during childbirth, as research has shown that women who experienced fear and lack of control or dissociation during birth are at risk of developing PTSD [8]. Moreover, the group that felt the most emotions of fault and failure was the ElCs group. Conversely, a study found no differences between the ElCs and VB groups regarding negative emotions such as dislike and disappointment [29]. Other studies found the birth experience to be equally positive among women who experienced ElCs and VB [2].

Women who went through unplanned birth procedures, such as VE or EmCs, reported higher levels of fear, lack of control, anger, conflict, fault, and failure than women who underwent planned birth procedures, such as VB or ELSC. It is noteworthy that negative emotions, including a lack of control or involvement in decision making, are strongly associated with negative birth experiences [30]. Similar to our study, another study found that women who have spontaneous vaginal birth reported the most positive emotions regarding their birth, while the women who had unplanned caesarean section reported the least positive emotions [23]. Unplanned birth procedures can be considered sudden, dangerous, overwhelming, and stressful events [30]. EmCs has been negatively associated with a mother’s emotions such as sadness and disappointment [29]. A phenomenological study revealed that unplanned birth procedure (such as EmCs) could raise emotions including fear, guilt, or anger, with lasting, prominent effects on women’s memories [28]. Unplanned births procedures are proven to increase fear and feelings of lack of control during the birth. These emotions can be a catalyst for postpartum complications, such as PTSD [8]. Unlike unplanned birth, planned birth procedures, either VB or ElCs, have been associated with a more positive experience in previous studies [2]. In order to lower negative emotions, such as the fear of childbirth, in women, it is necessary to identify the factors that contributed to them [1].

## 5. Conclusions

This study strives to assess women’s emotions during birth based on the mode of birth: VB, VE, ELSC, and EmCs. Furthermore, the study aims to evaluate if there were different emotions between primiparous and multiparous women’s during birth. Israel is a country that encourages having multiple children, so the social pressure on women is increasing, especially among primiparous women. It is important to be aware that during birth risk factors such as fear, lack of control, and dissociation might cause long-term damage. The early recognition of women at risk of negative emotions towards childbirth in clinical settings is important in order to help improve women’s healthcare during the pregnancy and after childbirth. Intrapartum interventions that emphasize communication, information sharing, and emotional and practical support during birth (especially among primiparous women) may be helpful. 

Women who underwent interventions, including EmCs, VE, and ELSC, might have experienced negative emotions during birth, such as fear, lack of control, and dissociation. Enhanced knowledge and understanding among caregivers would provide a vital step towards implementing effective plans to improve pregnant and postpartum women’s health. It is worth mentioning that postpartum women who demonstrated signs of mental stress, such as intrapartum dissociation, especially during birth with medical intervention, should provide postnatal support to prevent the development of PTSD. We conclude that particular attention to personal well-being must be given to women during childbirth, especially those who undergo unplanned birth procedures, as many who endure negative birth experiences later feel negative health-related consequences.

### Limitations and Recommendations for Future Research

There are three limitations to this research. First, our findings are based on a self-reporting survey, for which women with negative birth experiences are less likely to volunteer to fill out a questionnaire than those with more neutral or positive birth experiences. Therefore, a prospective study might be beneficial to neutralize this bias. Second, the study was conducted through the convenience sample method in Israel and has limited research sample representativeness. Therefore, it is recommended to investigate women with negative birth experiences in a large probability sample and in different countries. The third limitation is the applicate single tool (questionnaire) in this research. Therefore, further research should combine some tools, such as interviews, which might be helpful for identifying complex perceptions of women with negative birth experiences.

## Figures and Tables

**Table 1:** Background characteristics of participants.

Characteristics		*n* = 325	
		Frequency	Percent
Religion	Secular	89	28%
	Traditional	39	12%
	Religious	151	46%
	Orthodox	46	14%
Status	Single	3	1%
	Married	309	95%
	Divorced	13	4%
Education	High school	40	13%
	Higher Education	284	87%
Number of children	One	210	65%
	Two	90	28%
	Three+	25	7%
Parity	Multiparous	210	65%
	Primiparous	115	35%

**Table 2:** Differences in emotions based on primiparous and multiparous groups.

Emotions	Primparous *n* = 115 Mean (SD)	Multiparous *n* = 210 Mean (SD)	Mann-Whitney Z	*p*
Fear and lack of control	0.98 (0.72)	0.77 (0.65)	2.65	0.00
Anger and conflict	0.36 (0.49)	0.28 (0.41)	1.24	0.22
Intrapartum dissociation	0.82 (0.69)	0.43 (0.47)	5.68	0.00
Failure and fault	0.34 (0.64)	0.24 (0.47)	1.01	0.28

**Table 3:** Correlations between numbers of children birthed and negative emotions during last birth.

	Number of Children Birthed	Fear and Lack of Control	Anger and Conflict	Intrapartum Dissociation	Failure and Fault
Number of children birthed	1	−0.16 **	−0.056	−0.32 **	−0.11 *
Fear and lack of control		1	0.57 **	0.49 **	0.47 **
Anger and conflict			1	0.40 **	0.42 **
Intrapartum dissociation				1	0.31 **
Failure and fault					1

* *p* < 0.05; ** *p* < 0.00.

**Table 4:** Negative emotion differences during birth based on mode of birth.

Variables	VB *n* = 253 Mean (SD)	VE *n* = 21 Mean (SD)	ElCs *n* = 15 Mean (SD)	EmCs *n* = 36 Mean (SD)	Kruskal-Wallis (χ^2^)	*p*
Fear and lack of control	0.79 (0.63)	1.26 (0.79)	1.32 (0.90)	1.41 (0.80)	33.27	0.00
Anger and conflict	0.28 (0.41)	0.48 (0.61)	0.52 (0.50)	1.32 (0.90)	12.16	0.00
Intrapartum dissociation	0.63 (0.59)	1.1 (0.81)	0.82 (0.76)	0.77 (0.78)	11.22	0.01
Failure and fault	0.16 (0.36)	0.78 (0.77)	1.22 (1.05)	0.70 (0.86)	56.56	0.00

**Table 5:** Differences in negative emotions between women that experienced ‘planned’ birth versus ‘unplanned’ birth.

	Planned Birth Procedure *n* = 268 Mean (SD)	Unplanned Birth Procedure *n* = 57 Mean (SD)	Mann-Whitney (z)	*p*
Fear and lack of control	0.82 (0.66)	1.35 (1.33)	4.89	0.00
Anger and conflict	0.30 (0.42)	0.49 (0.63)	2.13	0.03
Intrapartum dissociation	0.64 (0.60)	0.87 (0.60)	1.85	0.06
Failure and fault	0.22 (0.49)	0.73 (0.82)	5.41	0.00

## Data Availability

Not applicable.
